# Natural Polyphenols for the Preservation of Meat and Dairy Products

**DOI:** 10.3390/molecules27061906

**Published:** 2022-03-15

**Authors:** Hammad Ullah, Yaseen Hussain, Cristina Santarcangelo, Alessandra Baldi, Alessandro Di Minno, Haroon Khan, Jianbo Xiao, Maria Daglia

**Affiliations:** 1 Department of Pharmacy, University of Naples Federico II, 80131 Naples, Italy; hammad.ullah@unina.it (H.U.); cristina.santarcangelo@unina.it (C.S.); alessandra.baldi.alimenti@gmail.com (A.B.); alessandro.diminno@unina.it (A.D.M.); 2 Lab of Controlled Release and Drug Delivery System, College of Pharmaceutical Sciences, Soochow University, Suzhou 215123, China; pharmycc@gmail.com; 3 Department of Pharmacy, Abdul Wali Khan University Mardan, Mardan 23200, Pakistan; haroonkhan@awkum.edu.pk; 4 Department of Pharmacy, Bashir Institute of Health Sciences, Islamabad 45400, Pakistan; 5 Department of Analytical Chemistry and Food Science, University of Vigo, 36310 Vigo, Spain; jianboxiao@uvigo.es; 6 International Research Center for Food Nutrition and Safety, Jiangsu University, Zhenjiang 212013, China

**Keywords:** food spoilage, food preservation, meat products, dairy products, polyphenols

## Abstract

Food spoilage makes foods undesirable and unacceptable for human use. The preservation of food is essential for human survival, and different techniques were initially used to limit the growth of spoilage microorganisms, e.g., drying, heating, salting, or fermentation. Water activity, temperature, redox potential, preservatives, and competitive microorganisms are the most important approaches used in the preservation of food products. Preservative agents are generally classified into antimicrobial, antioxidant, and anti-browning agents. On the other hand, artificial preservatives (sorbate, sulfite, or nitrite) may cause serious health hazards such as hypersensitivity, asthma, neurological damage, hyperactivity, and cancer. Thus, consumers prefer natural food preservatives to synthetic ones, as they are considered safer. Polyphenols have potential uses as biopreservatives in the food industry, because their antimicrobial and antioxidant activities can increase the storage life of food products. The antioxidant capacity of polyphenols is mainly due to the inhibition of free radical formation. Moreover, the antimicrobial activity of plants and herbs is mainly attributed to the presence of phenolic compounds. Thus, incorporation of botanical extracts rich in polyphenols in perishable foods can be considered since no pure polyphenolic compounds are authorized as food preservatives. However, individual polyphenols can be screened in this regard. In conclusion, this review highlights the use of phenolic compounds or botanical extracts rich in polyphenols as preservative agents with special reference to meat and dairy products.

## 1. Introduction

Fresh foods (meat, seafood, and horticultural products) are prone to foodborne disease outbreaks caused by pathogenic microbes, limiting their storage life [1]. Food spoilage is a metabolic process that makes foods undesirable or unacceptable for human use, due to alterations in their sensory characteristics. In some cases, such spoiled food may be safe for use and not cause illness, but changes in texture, taste, smell, and appearance lead to its rejection for consumption [2]. Thus, food preservation has been necessary for human survival since prehistory. In the past, techniques used for the preservation of food relied on the inactivation of spoilage microorganisms through drying, heating, salting, or fermentation [3]. The most important approaches in the preservation of food include a reduction in water activity in foods, the control of temperature, and the use of preservatives (sorbate, sulfite, or nitrite) and competitive microorganisms (lactic acid bacteria) [4]. In general, there are three types of preservatives: (1) antimicrobial agents, which prevent the growth of microorganisms that may cause serious illnesses (i.e., salmonellosis or botulism) and which are used in margarine and dressings, cheeses, bakery products, and dried fruit preparations; (2) antioxidants, which slow down the degree of oxidation and can be used in products containing unsaturated fatty acids that are more susceptible to oxidative reactions; (3) anti-browning agents, which are added to fruits and vegetables in order to prevent enzymatic browning [5]. The scientific literature has shown that artificial preservatives may, in certain cases, cause serious health hazards such as hypersensitivity, asthma, neurological damage, hyperactivity, and cancer [6].

In the modern era, many consumers prefer natural food preservatives over synthetic ones. The benefits of natural preservatives are endless, and these tend to be safer for use in comparison to synthetic preservatives [7]. Polyphenols are the largest group of plant secondary metabolites, containing benzene rings with hydroxyl moieties, and they can be divided into different chemical classes, including flavonoids, phenolic acids, lignans, tannins, and stilbenes. They are the most abundant phytochemicals found in dietary sources, possessing many pharmacological effects including antioxidant and antimicrobial activities [8,9]. The major sources of polyphenols include fruits or fruit juices (apple, grapefruit, orange, pineapple, and *Prunus* fruits), vegetables (broccoli, cabbage, carrot, cucumber, mint, spinach, tomato, and yellow onion), beverages (tea and coffee), and wine [10]. This group of compounds could play an essential role in the defense and protective mechanisms of botanicals [11]. They have potential use as biopreservatives in the food industry and have been extensively studied for the enhancement of the shelf life of perishable products. The use of phenolic compounds from natural sources is an interesting approach, as it allows the production of food without synthetic additives [12].

This review aims to highlight the potential role of natural polyphenols as potential preservatives in meat and dairy products, mainly focusing on their antimicrobial and antioxidant effects.

## 2. Oxidative and Microbial Spoilage of Food Components

Food proteins and lipids are highly exposed to oxidation, which affects their food safety and quality. Lipid and protein oxidation diminishes the shelf life and quality of food components, in addition to nutritional and sensorial deteriorations that in turn produce toxic substances [13]. Polyunsaturated fatty acid within food components contains double bonds that are the real initiators of the oxidation process. These double bonds react with atmospheric oxygen resulting in the production of free radicals and hydroperoxide [14]. Such oxidation is followed by protein oxidation, coagulation, polymerization, and protein carbonylation [15]. All these changes prevent natural proteolysis and protein solubility in food components. In addition, reduced pH, light, heat, and oxidative enzymes are other factors that promote the oxidation process [16].

In addition to the oxidative spoilage of food components, spoilage microorganisms are a major contributor to food quality degradation and safety issues. These microbes decrease the sensory and nutritional value of the food, as well as themselves being the cause of foodborne diseases. As an example, *Bacillus cereus* attacks noodles, pasta, and rice, resulting in the spoilage of these foods and the production of toxins. Milk is spoiled by psychrotrophic and mesophilic isolates [17]. Similarly, *Campylobacter coli* spoils unpasteurized milk and poultry products [18]. *Escherichia coli* has the tendency to spoil sprouts, unpasteurized milk, and ground meats [19]. *Clostridium botulinum* spoils meat and other foodstuffs, leading to compromised food safety, while *L. monocytogenes* affects soft cheeses, vegetables, and ready-made foods [20].

Filamentous fungi, usually referred to as molds, serve the beneficial function of recycling dead animals and plant remains; however, they also provide harsh consequences in terms of food spoilage, attacking their targets via airborne spores [21]. Molds can survive at a low pH (3–8) within foodstuffs and grow even with a limited supply of water [22]. *Penicillium*-based molds attack jams and margarines and lead to their spoilage. Similarly, *Byssochlamys* affects pasteurized juices, and its spores are highly temperature resistant. In addition, the *Aspergillus* species of molds are the spoilage microorganisms responsible for attacking and producing toxins in food items such as peanuts, grains, and beans [2].

Certain forms of microbial deterioration led to the spoilage of food components through alteration in their physicochemical properties. These adverse effects on food manifest as slime production, softening of texture, discoloration, and off-flavors. Animal-derived products including poultry, dairy, meat, and milk are spoiled by certain microbes including lactobacilli, *Brochotrix*, *Pseudomonas*, and Enterobacteriaceae [23]. Similarly, plant-derived products may be spoiled by certain molds and yeasts, e.g., *Penicillium*, *Candida*, *Aspergillus*, *Pichia*, and *Fusarium* species [24].

Fungi remain a primary concern for the spoilage of preserved foods, as they can proliferate even with a limited supply of water. Most fungi are also heat resistant, thus resisting cooking processes [25]. In addition, spores may survive within foodstuffs for a yet unknown period of time, dependent upon the availability of water [26]. A recent study found that sea salt may contain certain fungi including *Cladosporium*, *Aspergillus*, and *Penicillium*, which may spoil food and produce mycotoxins. These spoilage-causing and mycotoxigenic fungi have been found to favor limited-water environments [27]. 

The control and prevention of spoilage microorganisms relies on their proper detection within food. Certain food spoilage microorganisms have become resistant to conventional conservation methods for foodstuffs, and there is an urgent need for novel preservative techniques to shelter food components from microbial deterioration, to ultimately avoid food loss and comply with industrial demand. In addition, consumers do not favor the preservation of food using certain chemicals, thus providing an opportunity for researchers to discover natural sources for food preservation, in order to increase the shelf life of food products. A few natural preservation techniques are discussed in the next section. 

## 3. Natural Methods of Food Preservation

Food can be preserved using multiple techniques such as refrigeration and heating, although these techniques have certain drawbacks in the form of alteration of organoleptic features and nutrient loss. Natural preservatives are getting more attention in the food industry due to the drawbacks of artificial preservatives. Allyl isothiocyanate is a natural food preserving agent isolated from the essential oil of mustard and other species of the Brassicaceae family. It exhibits antimicrobial potential against food spoilage microbes. Due to its pungent taste, fast evaporation, and hydrophobic nature, its natural preservative potential is limited to certain applicable foodstuffs [28]. 

Essential oils are historically known for their aroma and microbicidal action. Apart from their property of modifying food flavor, they can also exhibit antimicrobial potential against foodborne pathogens, thus replacing chemical preservatives [29]. As natural food preservatives, essential oils can be used as natural food additives and as a bioactive component in packaging materials. Oregano essential oil is rich in thymol and carvacrol and is added to pork meat, resulting in inhibition of the growth of *L. monocytogenes* and an improvement in food flavor [30]. The food preserving capacity of citron oil (a kind of oil extracted from *Citrus medica* fruit) in a fruit-based salad was evaluated against *Salmonella typhi* and *L. monocytogenes.* The results indicate an outstanding antimicrobial potential against these species, confirming its use as a natural food preservation agent [31].

Peptides from animal sources have shown antimicrobial action against a wide range of pathogens associated with food components. Counts of multiple bacteria including *Serratia liquefaciens*, *Lactobacillus plantarum*, and *Zygosaccharomyces bailii* were successfully reduced in mayonnaise after the application of chitosan [32]. Alginates and carrageenan isolated from algae have shown an effective role in food preservation. These form nanocomposite films containing essential oils, which display antimicrobial action against spoilage microorganisms in food materials. Lactic acid bacteria favor controlled acidification, producing acids that in turn preserve important foodstuffs [33].

Some food components can also act as food preservative agents. Jellies, jams, and marmalades are composed of 70% sugar, which is itself not toxic to microbes, but rather absorbs water content from foodstuffs, thus restricting the growth of spoilage microorganisms [34]. Similarly, salt is used at a concentration of 20% in pickles. Salt triggers microbial cell plasmolysis through the induction of a high osmotic pressure. Dehydration of foodstuffs and the presence of chlorine ions are two further useful factors that salt provides in food preservation [35]. 

## 4. Phenols and Antioxidant Capacity

Phenols exhibit strong antioxidant potential due to their possession of aromatic rings with hydroxyl groups, acylated sugars, and organic acids in their structure. The antioxidant capacity provided by these moieties is due to the inhibition of free radical formation [36]. *Medicago minima* (L.) is a pasture legume that grows well around the world. A recent study revealed that a strong antioxidant capacity was observed for the phenols extracted from the roots, seeds, and leaves of *M. minima* [37]. Similarly, sorghum is a cereal exhibiting a high concentration of phenols that in turn are beneficial to human health due to their antioxidant potential [38].

Rosehips are fruits of species of the *Rosa* genus and are famous for treating digestive disorders and boosting the immune system. Rosehips contain phenols that exhibit strong antioxidant activity [39]. Such an antioxidant potential may attract the attention of consumers to use rosehips as potential functional foods. The antioxidant capacity of phenols can be measured indirectly via the estimation of their total phenolic contents. Olive oil and olives obtained from *Olea europaea* L. contain certain phenols including oleuropein, ligstroside, and verbascoside that exhibit strong antioxidant potential [40]. The *O. europaea* extracts obtained from the leaves are natural antioxidants with minimal toxicity, high-cost effectiveness, and improved bioavailability [12,41].

Polyphenols are also found in the seeds of grapes, in the form of gallic acid, monomeric catechin, and epicatechin. Many studies have revealed the antioxidant potential of these polyphenols [42]. Moreover, many fruits such as cherries, berries, and pomegranate and vegetables such as parsley, artichokes, and Brussels sprouts contain polyphenols with antioxidant activity [43]. In addition, the nutritional value of polyphenols is worth mentioning, as they protect oxidative chain proliferation via inhibition of lipids. Polyphenols from fruits and vegetables have strong antioxidant activity that detoxifies carcinogens and modifies metabolic activation [44].

During recent decades, the health benefits from the use and regular consumption of whole grains have been reported. In view of this, *Oryza sativa* L. and rice bran were studied to assess their phenolic content, and the results suggest that their secondary metabolites, i.e., ferulic acids, exhibit strong antioxidant potential [45]. Defatted rice bran contains phenols that have shown promising antioxidant potential and can be used as an alternative and cost-effective food additive [46]. A recent comprehensive review suggests that husk and straw from rice sources contain bioactive substances in the form of polyphenols, for which antioxidant activity appears to be the main mechanistic approach [47].

Catechins are abundant in tea, and these are well known for their antioxidant potential. Advanced techniques including ultrasound-assisted ultrafiltration and pulsed electric fields were used to extract polyphenolic compounds from tea, which have shown promising antioxidant activity [48]. Commercial teas were evaluated for their contents and antioxidant activity in a recent study comparing extractable and non-extractable polyphenols. The results indicate that both the antioxidant activity and contents of extractable polyphenols were found to be higher than those of the non-extractable polyphenols [49]. Theaflavin is another polyphenol extracted from black tea. Theaflavin exhibits powerful antioxidant potential as shown by its electro-analytical data [50].

Traditionally, food conservation is enabled by the antioxidant properties of herbs and spices. These properties are attributed to the presence of polyphenols. Mint, cinnamon, and clove contain polyphenols that provide antioxidant potential. The antioxidant activity of black garlic is also due to the presence of phenols in its composition [51]. All these findings show that polyphenols are a versatile class of phytochemicals that exhibit antioxidant potential with promising positive effects on human and animal health. Table 1 shows the antioxidant potential of some selected botanical sources having polyphenols as bioactive agents.

## 5. Phenols and Antimicrobial Activity

Chemical preservation in the food industry can lead to toxic side effects, and there is a need for suitable health friendly alternatives. Natural compounds (phytochemicals) are found in many foods as natural chemicals with antimicrobial potential. These compounds can be used as direct food antimicrobial agents, prolonging shelf life of food. The antimicrobial activity of plants and herbs is mainly due to the presence of phenolic compounds found in their extracts. Certain factors including pH, protein content, salt concentration, and temperature can affect the antimicrobial activity of these compounds [59]. In addition, food quality is affected by microbiological factors, control of which is essential for food preservation. The use of food additives of natural origin can overcome these issues associated with food preservation. 

In a study, 15 Mediterranean medicinal plants were evaluated for the antimicrobial activity of their phenolic contents. Bearberry showed the highest antimicrobial potential against Gram-positive bacteria, due to the highest concentrations of phenolic compounds [60]. Similarly, compounds extracted from herbs and spices have been explored as natural antibacterial additives. To this end, phenolic extracts were isolated from *Hibiscus sabdariffa* L. calyces and fractioned and analyzed against foodborne pathogenic bacteria. The results suggest that the phenolic extracts showed greater antimicrobial activity, providing extended shelf life in beef [61]. Another study explored the antimicrobial activity of phenolic contents from two edible spices *Aframomum melegueta* K. Schum. and *Afrostyrax lepidophyllus* Mildbr. Gallic and phenolic acids were found to be abundant in both species, with efficient antimicrobial potential. The results of the study also showed that non-communicable diseases could be managed by using the extracts from *Aframomum melegueta* in combination with those from *Afrostyrax lepidophyllus* as a natural source of antimicrobial agents [62].

The extracts and the essential oil from multiple types of oregano have been evaluated for their antimicrobial potential. The phenols, thymol and carvacrol, are active against Gram-positive bacteria such as *Staphylococcus epidermidis*, *Staphylococcus aureus*, and *Bacillus subtilis.* In addition to their activity against Gram-positive bacteria, these phenols were also found to be active against Gram-negative bacteria including *E. coli*, *Enterobacter cloacae*, and *Salmonella typhimurium* [63]. Similarly, a study evaluated the antimicrobial activity of cinnamon extract, in which the main constituents, cinnamaldehyde and eugenol, showed an efficient antibacterial activity against *E. coli* and *S. typhi* as measured through their respective zones of inhibition [64]. Phenols from *Cinnamon verum* J. Presl. bark showed antimicrobial action against *E. coli* and *Staphylococcus aureus* [65]. This indicates that these natural sources can be used as a natural source of antibiotics; however, more advanced studies are required. 

Berries from *Vaccinium meridionale* Sw. contain phenols such as anthocyanins and hydroxycinammic acid and have shown in vitro antimicrobial activity against Gram-positive and Gram-negative bacteria, thus making them potential candidates for the development of functional foods [66]. Similarly, the essential oil extracted from *Pistacia atlantica* Desf. was investigated for its phenolic content. The study showed that *P. atlantica* was abundant in phenols and showed outstanding antimicrobial activity against *E. coli* [67]. Soxhlet and maceration processes were used for the extraction of phenolic compounds from essential oils of *Ruta montana* (L.) The obtained phenolic compounds were tested against 12 strains of fungi and 28 strains of bacteria. The results showed strong antifungal and antibacterial activities for these phenolic compounds [68]. It suggests that *R. montana* is a valuable resource that exhibits cost-effective functional properties. This valuable byproduct could be used in cosmetics, food, and pharmaceutical industries. Other phenolic compounds from various sources are shown in Table 2, alongside their respective antimicrobial activities. 

## 6. Food Fortification with Phenols as Preservative Agents

Meat products are more vulnerable to lipid oxidation, which is often measured using the thiobarbituric acid reactive substances (TBARS) method. While synthetic antioxidants were initially used to prevent oxidation of lipids, natural sources have been found that might serve same purpose in meat [12]. The use of olive leaf extracts is a common strategy for the enrichment of food with phenol contents. The incorporation of olive leaf extract (with total phenolic contents of 45.2 mg gallic acid equivalent (GAE) kg^−1^) in cooked pork meat patties resulted in a significant delay in lipid oxidation and both primary (conjugated dienes and hydroperoxides) and secondary (malondialdehyde) oxidation products. Protein oxidation was also inhibited in a concentration-dependent manner by decreasing protein carbonyls and increasing protein sulfhydryls [81]. 

Bee pollen (0.2%) was found to be effective in retarding lipid peroxidation in pork sausage stored at 4 °C for 30 days, showing significantly lower values of TBARS compared to control [82]. The percentage decrease in TBARS values was highest in storage after 10 days. The storage life of pork nuggets increased from 21 to 35 days with the incorporation of *Averrhoa carambola* L. fruit juice extract, in comparison to pork nuggets without the extract. The TBARS values of pork nuggets were found to be lower with fruit juice extract (4% and 6%) during 35 days of storage [83]. The addition of green tea extract in hamburger showed a reduction in TBARS values during the 8-day storage period. The effect of tea was increased in a combination of green tea extract with chitosan, as the resistance to lipid oxidation and microbial deterioration was significantly increased [84].

In another study, pork sausages fortified with a chitosan-film incorporating green tea extract showed decreased changes in color, texture, thiobarbituric value, microbial growth, and sensory characteristics, when compared to control (chitosan alone or green tea extract without chitosan). Successful inhibition of microbial growth (yeasts and molds, and lactic acid bacteria) and lipid oxidation was observed in refrigerated pork sausages, suggesting that the incorporation of green tea extract into chitosan may enhance the antimicrobial and antioxidant properties of the film, and thus, maintain the prolonged shelf-life of the sausages [85].

The addition of different spice extracts (*Syzygium aromaticum* (L.) Merr. and L.M. Perry, *Cinnamomum cassia* (L.) J. Presl., *Origanum vulgare* L., and *Brassica nigra* (L.) K. Koch) with high total phenolic content to raw chicken meat demonstrated an effective prevention against microbial growth and lipid peroxidation. The total phenolic contents ranged from 14.09 to 24.65 GAE/g. Samples with *Syzygium aromaticum*, *C. cassia*, and *Origanum vulgare* extracts exhibited a greater reduction of bacterial counts (lactic acid bacteria and Enterobacteriaceae) and TBARS concentrations than control, with a positive increase in sensorial properties such as color and odor over a storage period of 4 °C for 15 days [86]. This kind of fortification of raw meat with vegetable extracts can be effective for preservation, while providing lower TBARS values during storage for 20 days at temperatures ranging from 4 to 20 °C [87].

In addition to its capacity to delay lipid and protein oxidation, pomegranate peel extract can also be used for its melanosis-inhibitory activity during storage of Pacific white shrimp in refrigerators, with a decrease in mesophilic, psychrophilic, lactic acid bacteria, and Enterobacteriaceae counts [88,89]. Natural phenols derived from barley husks slow down lipid hydrolysis and increase the oxidative stability of salmon fish, as determined by peroxide value, conjugated dienes, conjugated triene hydroperoxides, free fatty acids, totox values, thiobarbituric acid index, and *p*-anisidine values [90]. Barley husks are quite rich in phenolic acids (p-coumaric acid, trans-ferulic acid, and syringic acid), as revealed by LC-MS analysis [91]. Barley husks also slow down lipid hydrolysis and oxidation (reflected by significant decreases in lipid hydrolysis and TBARS values) in blue shark (packaged in a film) during storage at −20 °C for 6 months [92]. 

Several studies suggest that the packaging application of films incorporated with natural antioxidants improves food stability (from aqueous to fatty food products) throughout storage.

Barbosa-Pereira et al. developed active antioxidant films with natural antioxidants (brewery residual stream extract and commercial rosemary extract) using a coating technique, and these films increased the oxidative stability of beef during refrigeration, reducing lipid oxidation up to 80% in comparison with the control [93]. Incorporation of catechin and quercetin into ethylene–vinyl alcohol copolymer films successfully improved the antioxidant protection of packaged food, with the most significant results being observed with catechin [94]. Similar results were observed with green tea extract incorporated in ethylene–vinyl alcohol copolymer films [95].

Active films treated with oregano significantly protected lamb against oxidation and microbial spoilage, as seen in the improvement in metmyoglobin formation, TBARS values, instrumental color, psychrotrophic aerobic flora counts, and sensory discoloration [96]. When applied to the packaging of ground beef stored at 3 °C, multilayered polyethylene films with incorporated grapefruit seed extract demonstrated a reduction of growth rates of numerous microbes including *Escherichia coli* IFO 3301, *Staphylococcus aureus* IFO 3060, and *Bacillus subtilis* IFO 12113 [97]. They also slowed down the chemical changes in packaged beef during storage.

Chouchouli et al., reported that yogurt fortified with grape seed extracts (rich in polyphenols) contained more bioactive compounds, with higher antioxidant and antiradical activities [98,99]. Similarly, oat-bran-fortified raspberry probiotic dairy drinks exhibited increased antioxidant effects, owing to a higher phenolic content [100]. Strawberry polyphenol extract–fortified stirred dahi (a traditional fermented dairy product prepared by lactic acid fermentation of milk) resulted in a seven-fold increase in the antioxidant activity while pH, acidity, water-holding capacity, and viscosity remained comparable with the control [101]. The addition of grape pomace powders to semi-hard (Italian Toma-like) and hard cheeses (cheddar) resulted in increased total phenolic contents and radical scavenging activity, while no variation was observed in the microbial counts and physiochemical parameters [102]. Tseng and Zhao stored grape-pomace-fortified yogurt for 3 weeks at 4 °C and observed an increase in pH and decrease in viscosity without alterations in lactose concentrations [103]. In addition, grape pomace also reduced the peroxide values during storage with advantages in oxidative stability. Polyphenol-enriched dairy products developed by incorporating black carrot concentrate demonstrated enhanced antioxidant activities with increased total phenolic contents [104]. The storage study showed that yogurt can be stored for up to 5 days, ice cream for more than 60 days, and buttermilk for up to 10 days with excellent stability attributes.

The addition of dry rosemary to cottage cheese resulted in the highest antioxidant and antimicrobial effects due to high content of caffeic acid, rosmarinic acid, flavones, and phenolic diterpenes [105]. It was shown to limit the growth of foodborne pathogens including *Staphylococcus aureus*, *Escherichia coli*, *Listeria monocytogenes*, and *Salmonella typhimurium* during 3-day storage at 4 °C. Polyphenols contained in dry extracts from plants such as dill, parsley, garlic, and red sweet peppers were also tested in the same study and showed considerable antioxidant and antimicrobial activities, which was attributed to high polyphenolic contents in the final dried products. However, rosemary showed the highest antioxidant activity with a FRAP value of 17.1–26.4 mmol per 100 g, followed by dill, parsley, red sweet peppers, and garlic. *Citrus aurantium* L. flower extract containing total phenolic and flavonoid contents of 81 and 46 mg/g, respectively, was studied in yogurt stew during storage for 28 days at 4 °C [106]. The extract was shown to inhibit the growth of *Pseudomonas aeruginosa*, *E. coli* O157:H7, *Bacillus cereus*, and *Staphylococcus aureus*. The extract showed significant antioxidant potential where the IC_50_ value for DPPH assay was calculated as 41.6 μg/mL while the IC_50_ value for control (butylated hydroxytoluene) was 18.8 μg/mL. Similarly, a FRAP assay showed a reducing power of the extract of 18.47 mmol Fe^2+^/mass. Anisidine value, peroxide value, protein carbonyls value, and conjugated diene value indicated that *Citrus aurantium* reduced protein and lipid oxidation products in yogurt stew during storage. *Punica granatum* L. rind extract demonstrated significant lipid oxidative stability and antimicrobial effects when added to cheese stored for 28 days at 4 °C, suggesting its potential use as a natural preservative in dairy products [107]. *Punica granatum* extract exhibit a significant decrease on TBARS (mg malonaldehyde/kg) and free fatty acid (% oleic acid) values. In addition, considerably lower values were observed for total plate count (log cfu/g), yeast and mold count (log cfu/g), and psychrophilic bacterial count (log cfu/g) in samples with added *P. granatum* extract. Organic cottage cheese flavored with Argentinean oregano essential oils (Cordobes, Compacto, Mendocino, and Criollo) was tested for the quality of storage and shelf-life at thermal storage for 30 days by Asensio and colleagues [108]. The samples flavored with thymol and Cordobes essential oil presented reduced conjugated dienes (15.53 and 15.94, respectively) as compared to 17.54 for the control sample. Samples flavored with Cordobes, Criollo, and Compacto essential oils exhibited reduced saturated/unsaturated fatty acid ratios than the control (1.62, 1.68, and 1.67, respectively). A significant low production of organic acids during storage was found in the samples flavored with Cordobes and Compacto essential oils.

## 7. Conclusions

Polyphenols are plant secondary metabolites with well-established health benefits. Due to an increasing demand for minimally processed food, polyphenols have drawn great interest as possible alternative preservative agents, potentially aiding oxidative stability and providing antimicrobial effects. Numerous studies have demonstrated that plant extracts rich in phenolics are effective agents in preventing microbial growth and oxidative processes in meat and dairy products, thus increasing their stability and storage life. Since no pure phenolics are authorized as food preservatives, direct incorporation of botanical extracts (rich in polyphenols) into perishable foods can be considered [108] In addition to their uses as antimicrobial and antioxidant agents, natural phenols can also be used as anti-browning agents. Individual phenolic compounds should also be screened for their possible uses as preservative agents in food products susceptible to spoilage by multiple mechanisms.

## Figures and Tables

**Table 1:** Antioxidant potential of some selected botanical sources.

Plant Species	Part Used	Extract	Total Phenolic Contents	Antioxidant Activity	References
*Rosa canina* L.	Fruits	50% ethanol	69.4 mg GAE/g dry weight	DPPH: 295 mM TE/g ABTS: 368 mM TE/g FRAP: 390 mM TE/g	[52]
*Olea europaea* L.	Leaves	Methanol	1.60 mg GAE/g dry weight	DPPH: IC_50_ 34.58 (µg/mL) ABTS: 37.93 g Trolox/100 g FRAP: 30.1 g Trolox/100 g	[53]
*Vitis vinifera* L.	Pomace	Methanol	74.75 mg GAE/g dry weight	ABTS: 485.42 µM TEAC/g DPPH: 505.52 µM TEAC/g FRAP: 249.46 µM TEAC/g	[54]
*Punica granatum* L.	Peel powder extract	Methanol	54.84 mg GAE/g	DPPH: 88.82% inhibition TBARS: 64.49% inhibition FRAP: 0.99 mM TE/g	[55]
*Petroselinum crispum* (Mill.) Fuss	Leaves	Distilled water	12.49 mg GAE/g dry weight	DPPH: EC_50_ 15.50 mg/mL FRAP: 189.8 mM Fe(II)/mg	[56]
*Oryza sativa* L.	Rice bran	10% glycerol	523.2 mg GAE/100 g dry weight	DPPH: 42.9% inhibition ABTS: 97.92% inhibition FRAP: 0.08 mM TE/mL	[57]
*Camellia sinensis* L.	Fruit peel extract	75% ethanol	53.12 mg GAE/g dry weight	DRAP: EC_50_ 1217 µg/mL ABTS: EC_50_ 849 µg/mL	[58]

Gallic acid equivalent (GAE); Trolox equivalent (TE); Trolox equivalent antioxidant capacity (TEAC); 2,2-diphenyl-1-picrylhydrazyl (DPPH); 2,2′-azino-bis-3-ethylbenzothiazoline-6-sulfonic acid (ABTS); ferric reducing antioxidant power (FRAP).

**Table 2:** Phenolic compounds and their antimicrobial activity.

Compound	Source	Antimicrobial Activity	Reference
Chlorogenic acid, caffeic acid	*Melipona beecheii* honey	*Staphylococcus aureus* and *Escherichia coli*	[69]
Chlorogenic acid	*Chaenomeles japonica*	*Enterococcus faecalis*	[70]
Flavonoids (luteolin, apigenin, quercetin, acacetin), phenolic acids (coumaroyl acid, hydroxybenzoic acid, rosmarinic acid, salvianolic acid, lithospermic acid)	*Satureja montana*, *Origanum majorana*	*Candida tropicalis*, *Staphylococcus aureus*, *Enterococcus faecalis*, and *Klebsiella pneumoniae*	[71]
Phenolic acid, chlorogenic acid	*Tilia cordata*	*Candida glabrata*, *Streptococcus pyogenes*, *Staphylococcus aureus*, and *Streptococcus mutants*	[72]
Gallic acid, chlorogenic acid	*Opuntia littoralis*	*Staphylococcus aureus*, and *Candida albicans*	[73]
Phenolic acids, stilbenes	*Punica granatum*	*Escherichia coli*, *Staphylococcus aureus*, and *Salmonella typhi*	[74]
Tannins, stilbenes	*Marsilea minuta*	*Pseudomonas aeruginosa* and *Klebsiella pneumonia*	[75]
Proanthocyanidins	Grape seed extract	*Listeria monocytogenes*	[76]
Proanthocyanidins	Cranberry	*Candida albicans*	[77]
Proanthocyanidins	Peanut	*Bacillus cereus*	[78]
Anthocyanins	Wild blueberries	*Salmonella enteritidis*, *Listeria monocytogenes*, *Vibrio parahaemolyticus*, and *Staphylococcus aureus*	[79]
Anthocyanins	*Aronia melanocarpa*	*Escherichia coli*	[80]

## Data Availability

Not applicable.
