# Correction: Ullah et al. Natural Polyphenols for the Preservation of Meat and Dairy Products. *Molecules* 2022, *27*, 1906

**DOI:** 10.3390/molecules30010053

**Published:** 2024-12-27

**Authors:** Hammad Ullah, Yaseen Hussain, Cristina Santarcangelo, Alessandra Baldi, Alessandro Di Minno, Haroon Khan, Jianbo Xiao, Maria Daglia

**Affiliations:** 1 Department of Pharmacy, University of Naples Federico II, 80131 Naples, Italy; hammad.ullah@unina.it (H.U.); cristina.santarcangelo@unina.it (C.S.); alessandra.baldi.alimenti@gmail.com (A.B.); alessandro.diminno@unina.it (A.D.M.); 2 Lab of Controlled Release and Drug Delivery System, College of Pharmaceutical Sciences, Soochow University, Suzhou 215123, China; pharmycc@gmail.com; 3 Department of Pharmacy, Abdul Wali Khan University Mardan, Mardan 23200, Pakistan; haroonkhan@awkum.edu.pk; 4 Department of Pharmacy, Bashir Institute of Health Sciences, Islamabad 45400, Pakistan; 5 Department of Analytical Chemistry and Food Science, University of Vigo, 36310 Vigo, Spain; jianboxiao@uvigo.es; 6 International Research Center for Food Nutrition and Safety, Jiangsu University, Zhenjiang 212013, China

In the original publication [1], Reference [49] was wrongly inserted due to some technical issues in the online system. We have thus deleted it: “Vickers, N.J. Animal communication: When I’m calling you, will you answer too? *Curr. Biol.* **2017**, *27*, R713–R715”. With this correction, the order of the references has been adjusted accordingly.

According to the suggestions from the Academic Editors, several terms and sentences were revised:

1. “spoiling microbes” was changed to “spoilage microorganisms”; 

2. “The most important approaches in preservation of food are the decrease of the presence and effects of water” was changed to “The most important approaches in the preservation of food include a reduction in water activity in foods”;

3. “Oxidation reduces the shelf life of food components” was changed to “Lipid and protein oxidation diminishes the shelf life and quality of food components”;

4. “microbial spoilage is a highly significant source of food spoilage” was changed to “spoilage microorganisms are a major contributor to food quality degradation and safety issues”.

The authors state that the scientific conclusions are unaffected. This correction was approved by the Academic Editor. The original publication has also been updated.
